# Translation and validation of the Child and the Adolescent HARDSHIP (Headache-attributed restriction, disability, social handicap and impaired participation) questionnaire into Danish language

**DOI:** 10.7717/peerj.1927

**Published:** 2016-04-14

**Authors:** Jens Erik Jorgensen, Kate A. McGirr, Hanne Oertved Korsgaard, Michael S. Rathleff

**Affiliations:** 1 Physiotherapy Private Practice, Aalborg, Denmark; 2 Department of Occupational Therapy and Physiotherapy, Aalborg University Hospital, Aalborg University, Aalborg, Denmark; 3 Research Unit for General Practice in Aalborg and Department of Clinical Medicine, Aalborg University, Aalborg, Denmark

**Keywords:** Headache, Adolescent, Burden, Child, Migraine, Denmark

## Abstract

**Background.** The prevalence of headaches among children and adolescents varies considerably between countries. This may be due to a lack of appropriate instruments to capture the prevalence. The purpose of this study was to translate the Child and Adolescent HARDSHIP questionnaires from English into Danish language, conduct cross-cultural adaptation, face validation by cognitive interviewing and conduct a pilot study exploring time requirements.

**Methods.** The questionnaire was translated using the guidelines proposed by “The Global Campaign to Reduce the Burden of Headache.” A total of 25 children from 6 to 12 years of age completed the questionnaire with 24 h between test and retest to assess reliability. A total of 169 children and adolescents from 6 to 17 years of age completed the translated questionnaire to assess time requirements for completing it.

**Results.** Only minor discrepancies were observed in the translation process. Test-retest reliability of the translated questionnaire showed substantial agreement (kappa: 0.65–0.78). The questionnaires were completed within 30 min (age 6–11 years of age) and within 15 min (age 12–17 years of age) respectively.

**Discussion.** No major problems were observed in the forward translations of the questionnaires. The face validation prompted no major changes in the questionnaire. The face-to-face interviews showed that pupils of different ethnic backgrounds than Danish and pupils in the age group of 6–11 had more difficulty in understanding a minority of the questions. The Danish Child and Adolescent HARDSHIP questionnaire therefore complies with the intentions of the originators, aiming at a maximal completion time of 45 min and in comparison with actual completion time. The test-retest study showed substantial agreement between test and retest in the headache, migraine and MOH domains and questions referring to time.The Child and Adolescent HARDSHIP questionnaire, includes a section specifically recording a four-week period.The Child and Adolescent HARDSHIP questionnaire is intended to measure burden of headache in large populations and there is therefore no need to reflect the states of individuals. We therefore find the substantial reliability of the Danish version of the Child and Adolescent HARDSHIP questionnaire to be adequate, without supplementing with a diary. The pilot study indicates that headache is a major problem among children and adolescents in Denmark. A total of 95.3% of the pupils have experienced headache in their life, 76.6% during the last year. A total of 14% left school early because of their headache and 14.9% missed school during the last four weeks, due to headache. 49.2% have experienced headaches during the last week and 47% describe the headache as “quite bad.” A total of 24.1% have taken pills or medicine during the last week due to headache. This pilot study clearly demonstrates the need to investigate the burden of headache among Danish schoolchildren as it seems to have a profound effect on their lives.

## Introduction

The global burden of headache is a significant health concern. Headache disorders rank third among the worldwide causes of disability, measured in years of life lost to disability (Steiner et al., 2015). In Europe, the total annual financial cost of headache amongst adults aged 18–65 years is estimated to be €173 billion (Linde et al., 2012).

The prevalence of headache in children and adolescents varies considerably. The prevalence of migraine is between 3% and 11% while the prevalence of tension headache ranges from 10% to 24% (Antonaci et al., 2014). A review by Wöber-Bingöl (2013), covering 64 studies on headache and migraine in children and adolescents reported a prevalence of 54.4% for any type of headache and 9.1% for migraine (Wöber-Bingöl, 2013). Among 12 to 15 year old adolescents, Straube et al. (2013) found a prevalence of 66 to 71% for at least one episode of headache in the past three months, and 33 to 40% for weekly headaches. This makes headache the most common neurological symptom and commonest manifestation of pain in childhood, with a high risk of developing chronic manifestations into adulthood (Antonaci et al., 2014). The wide range of prevalence may be the result of the different methods and instruments used to capture the prevalence (Kernick, Reinhold & Campbell, 2009).

The Global Campaign against Headache has launched initiatives to combat this issue and standardize the methods and instruments used in studies on headache. Patient reported outcome measures (PROMs) provide an objective measure of a subjective construct: that is, an individual’s experiences and concerns in relation to their health, health care and quality of life (Haywood, 2007). The appropriate selection of an outcome measure should be guided by evidence of measurement properties, for example reliability, validity and responsiveness, and practical properties, such as patient acceptability and feasibility (Haywood, 2006). One of the requirements is a survey instrument with good cross-cultural validity (Peters & Passchier, 2006).

The Global Campaign against Headache has recommended utilizing questionnaires such as the Child and Adolescent HARDSHIP questionnaires in order to provide valid estimates of the prevalence and impact of headaches among children and adolescents. However, the Child and Adolescent HARDSHIP questionnaires does not exist in Danish.

The purpose of the current study is to translate the Child and Adolescent HARDSHIP questionnaires from English into the Danish language and conduct a cross-cultural adaptation to a Danish context including face validation by cognitive interviewing. The study also had the aim of completing a pilot study with a larger group of pupils within the target age group of 6–17 to test the time requirements for completing the questionnaires.

## Materials and Methods

### Ethical approval

Before initiating the study, the authors contacted the Ethics Committee for North Jutland, Denmark. The Ethics committee stated that no approval was necessary, as the study did not include an intervention. Oral and informed consent was obtained from all participants. The study complied with the Declaration of Helsinki.

### The Child and Adolescent HARDSHIP questionnaire

The Headache-Attributed Restriction, Disability, Social Handicap and Impaired Participation (HARDSHIP) questionnaire (Steiner et al., 2014) is designed for application by trained lay interviewers. HARDSHIP is a modular instrument incorporating demographic enquiry, diagnostic questions based on ICHD-3 beta criteria, and enquiries into components of headache-attributed burden. HARDSHIP has already demonstrated validity and acceptability in multiple languages and cultures (Steiner et al., 2014). A similar questionnaire has been developed to assess the global estimation of burden of headache in children and adolescents, The Child and Adolescent HARDSHIP questionnaire (Wöber-Bingöl et al., 2014). It can be used to capture the prevalence of headache, the impact of headache on their lives and as a diagnostic tool to differentiate between tension type headache (TTH), migraine and Medication Overuse Headache (MOH) type of headache. The Child and Adolescent HARDSHIP questionnaire consists of a total of 44 questions: one to record the date; two demographic questions; two screening questions for headache prevalence; 10 headache diagnostic questions (related to headache characteristics and associated symptoms); four questions enquiring into the frequency of headache and the use of abortive medication, four questions related to activity loss; three questions related to headache yesterday; six questions referring to other aspects of headache-attributed burden; 12 questions about quality of life. The questionnaire is acceptable, valid and feasible for a global assessment of the burden of headache in children and adolescents (Wöber-Bingöl et al., 2014).

### Translation procedure for the Child and Adolescent HARDSHIP questionnaire

The translation procedure was done in accordance with the guidelines proposed by the Translation Working Group for the translation of documents produced by Lifting The Burden (LTBT, 2011). The process was supplemented with the guidelines produced by Beaton et al. (2000) and Beaton et al. (2007), the principles of good practice proposed by ISPOR (Wild et al., 2005) and translating instruments for cross-cultural studies in Headache Research (Peters & Passchier, 2006).

The procedure was as follows:

(1) Permission to translate the Child and Adolescent HARDSHIP questionnaire. The original questionnaires and translation guidelines were obtained from the developers by e-mail (Steiner et al., 2014; Wöber-Bingöl et al., 2014).

(2) *Forward translation.* Two independent bilingual Danish residents (T1 and T2), with Danish as their birth languages, translated the questionnaires into Danish. T1 was aware of the concepts being examined, whereas T2 was not (Beaton et al., 2000; Peters & Passchier, 2006). A translation coordinator, overseeing but not carrying out the translation, was selected according to the criteria stipulated by LTBT (2011).

(3) *Reconciliation.* Separate meetings were held with each of the translators (T1 and T2) to address discrepancies in the forward translations. A written report documented issues in relation to the translation process (Beaton et al., 2000; Peters & Passchier, 2006). If there were any unresolved queries, the original developers were contacted to resolve the query (Steiner et al., 2014; Wöber-Bingöl et al., 2014).

4) The agreed-upon, forward-translated versions (T12) of the Danish questionnaire was hereafter translated back to English by two Danish residents whose birth language was English (BT1 and BT2). BT1 was a research physiotherapist, whereas BT2 had no medical background. BT1 and BT2 were both blinded to the purpose of the questionnaire and had not seen the original Child and Adolescent HARDSHIP questionnaires (Beaton et al., 2000; Peters & Passchier, 2006).

(5) The translated versions were compared to the original versions to ensure conceptual equivalence; the remaining discrepancies and ambiguities were resolved among the project manager, BT1, and BT2. The back-translation was forwarded to the original author with a request to compare the original and back-translated versions and assess their conceptual equivalence (LTBT, 2011).

(6) *Harmonisation.* The harmonisation group consisted of the back- and forward translators, a research physiotherapist and a language competent physiotherapist. The project manager communicated with each member of the harmonisation group individually by email, due to geographic differences. Subsequently, through group-email correspondence, the team agreed upon the harmonised versions of the Danish Child and Adolescent HARDSHIP questionnaires. All members of the harmonisation group approved the translated versions.

(7) A test-retest study was conducted in a primary school. The ages of the participants ranged from 6 to 12 years of age. The objective of the pilot study was to evaluate the time needed for completion of the questionnaire within the different age groups and the need for help and mediation of the participants by the teachers. The answers were anonymous. The age group selected was in expectation of this being the age group where additional help in understanding the questionnaire would be most needed.

(8) A pilot study was conducted at a primary school. The ages of the participants ranged from 6 to 17 years of age. The objective of the pilot study was to evaluate the time needed for completion of the questionnaire within the different age groups and the need for help and mediation of the participants by the teachers. The answers were anonymous. The age group selected was in accordance with the target age of the questionnaire.

(9) Cognitive interviews were carried out at both schools. 12 children within the target ages of 6–17, were interviewed while completing the questionnaire (LTBT, 2011). The interviews varied between 30 and 45 min. The children were all affected by headache and native speakers of the Danish language. The interviewer was a qualified teacher, well-trained in the method of cognitive interviewing and proficient in interviewing children. This procedure provided a validity check and the possibility to amend potential errors and problems and to minimise future response errors and non-response (LTBT, 2011; Kuusela & Paul, 2000; Wild et al., 2005).

(10) The results from the cognitive interviews were reviewed and a final translated version of each questionnaire was prepared. Any issues relating to the interpretation of the questionnaires were documented in writing (LTBT, 2011; Kuusela & Paul, 2000; Wild et al., 2005).

(11) A lay person with no medical or research background with good linguistic understanding (a school teacher) assessed the back-checked consensus-based translation for readability, grammatical correctness and cultural suitability. Queries were documented and sent to the co-ordinator (LTBT, 2011).

12) The final translated versions were proofread and checked for errors of spelling and grammar, then the layout was finalised by an expert committee consisting of the project manager, two research physiotherapists and a qualified teacher (LTBT, 2011; Beaton et al., 2000).

(13) A final report documenting the translation procedure was produced (LTBT, 2011).

### Test-retest reliability of the Danish Child and Adolescent HARDSHIP questionnaire-DK

The Child and Adolescent HARDSHIP questionnaire was completed twice with 24 h between test and retest by 25 children aged 6–12 to assess test-retest reliability. The 24-hour period was chosen as headache due to practical reasons. The study sample was intentionally heterogeneous as the Child and Adolescent HARDSHIP questionnaire is intended for use in multiple conditions as suggested by the originator (Kuusela & Paul, 2000). The questionnaire was completed online using Google Docs within the school’s intranet system. The response rate was 100% at both test and retest. See Table 1 for participant characteristics.

**Table 1:** Basic characteristics of the pupils included in the study.

Basic pupil characteristics			
	Pilot study	Think aloud	Test–retest
Pupils included (*n*)	169	12	26
Gender			
Male (%)	82 (48.5%)	5 (41.7%)	12 (46.2%)
Female (%)	87 (51.5%)	7 (58.3%)	14 (53.8%)
Mean age (±SD)	11.2 (±2.5)	7.6 (±1.2)	10.7 (±1.9)

### Pilot study to assess the time requirements for completing the questionnaire

109 children age ranging from 6 to 11 years of age completed The Child HARDSHIP questionnaire and 60 children age ranging from 12 to 17 completed The Adolescent HARDSHIP questionnaire. The objective of the pilot study was to evaluate the time needed for completion of the questionnaire within the different age groups and the need for help and mediation of the participants by the teachers. The answers were anonymous. The age group selected was in accordance with the target age of the questionnaire. The pupils received the instruction of reading the questionnaire to answer the questions to the best of their ability. The teacher present clarified any queries as to the understanding of the questions.

### Cognitive interviews

Cognitive interviews of 12 pupils with headache were performed to assess the harmonised versions in a local primary school. (LTBT, 2011; Beaton et al., 2007). A teacher, well trained in the method of cognitive interviewing, conducted the interviews. The pupils interviewed were within the target age groups of the questionnaire and randomly selected in six different school grades. (Table 1) The interview method employed was a “Thinking aloud” study (Someren, Barnard & Sandberg, 1994) and varied between 10 and 35 min. During the course of a usability study, the children were asked to verbalise their thoughts and opinions while completing the questionnaire. This procedure provided a validity check and the possibility to amend potential errors and problems and to minimise future response errors and non-response (Kuusela & Paul, 2000).

### Statistics

Demographics of the included pupils are given as mean and the standard deviation. Questions 4–15 are answered on a categorical scale. Questions 16–22 are based on questions relating to the number of days and therefore numerical data. Question 23 consists of two categories, categorical and numerical data. Question 27 numerical data. Questionlating to the number of days and therefore numerical data. Question 23 consists of two categories, categoric while completing the questionnaire. This procedure provirical data. The interpretations of kappa values follow  Landis & Koch (1977) and Streiner, Norman & Cairney (2014). The test-retest reliability of the total number of days was analysed using an Intraclass correlation (ICC 2.1).

## Results

### Translation

No major problems were observed in the forward translations of the questionnaires and only minor discrepancies were found by the originator, primarily consisting of differences in the choice of synonyms and use of prepositions. Examples are: the expression “official relations” in the original version has specific meaning which is not captured by “cooperation” in the translated version, the expression “exercise” in the original was not covered by the expression “movement” in the translated version, the original was “things” (i.e., some things), which is not the same as “anything” (i.e., all things). These discrepancies were corrected and approved by the originator (Kuusela & Paul, 2000).

### Cognitive interviews

The interview with the pupils prompted no major changes in the questionnaire. Only a few minor changes were made (see Supplemental Information 4 for a complete list). In the age group between 6 and 11 a problem was encountered relating to time recollection. The time span of 4 weeks and 1 year was difficult for the youngest. This age group also showed difficulty in comprehending longer sentences. In the age group 12–17 there were no difficulties, and the pupils assessed the questionnaire as understandable.

### Pilot study

Table 1 showing the characteristics of the included participants in the three studies.

The pilot study included 169 pupils between 6 and 17 years of age. Completion time of the questionnaire was between 3 and 30 min for the youngest and between 3 and 15 min for the oldest age group. Pupils of different ethnic backgrounds had in general more difficulty in understanding a minority of the questions. Pupils in the 6–11 age group, needed mediation to clarify a minority of the questions. The pilot study indicates that headache is a major problem in these age groups. A total of 95.3% of the pupils have experienced headache in their life, 76.6% during the last year. A total of 49.2% have experienced headaches during the last week, 47% describe the headache as quite bad. A total of 24.1% have taken pills or medication during the last week due to headache. A total of 14% have left school early during as a result of their headache and 14.9% missed school during the last four weeks, due to headache.

### Reliability

“The test-retest study showed substantial agreement between test and retest in the headache, migraine and MOH domains (absolute agreement = 76.9–96.2% and kappa = 0.65–0.78). The sections of the questionnaire referring to time, indicated almost perfect agreement. ICC 0.85 (95% CI [0.67–0.93]).” Question 23 showed almost perfect agreement (absolute agreement 95.7 % and kappa = 0.78) (Table 2).

**Table 2:** Test-retest values of the HARDSHIP questionnaire.

Test-retest study. Kappa, agreement percentage and Intraclass Correlation Coefficient (ICC)			
Domains	Absolute agreement %	Kappa	Intraclass Correlation Coefficient (ICC)
Questions			
Screening	96.2	0.78	
Q 4–5			
Diagnostic	86.8	0.77	
Q 6–15			
Impact headache on life the past 2 and 4 weeks			0.85
Q 16–22			
Impact parents: loss of work due to child’s headache	95.7	0.78	
Q 23			
Impact yesterday	86.5	0.70	
Q 24–26			
Impact last 4 weeks	82.5	0.73	
Q 27–32			
Quality of life in general	78.9	0.70	
Q 33–44			

## Discussion

An internationally recommended translation procedure was used to produce a Danish version of the Child and Adolescent HARDSHIP questionnaires, and a rigorous translation methodology was used to ensure the questionnaires were translated and adapted into a Danish context. Cognitive interviews were used to provide a face validation of the translated versions of the Child and Adolescent HARDSHIP questionnaire, thereby minimizing non-responses and response errors (LTBT, 2011; Wild et al., 2005).

No major problems were observed in the forward translations of the questionnaires. The face validation prompted no major changes in the questionnaire. The face-to-face interviews showed that pupils of different ethnic backgrounds than Danish and pupils in the age group of 6–11 had more difficulty in understanding a minority of the questions.

The Danish versions were completed within 30 min for the age group of 6–11, and within 15 min for the age group of 12–17. In the original questionnaire (Wöber-Bingöl et al., 2014) the average time needed to complete the questionnaire was within 9 min (range 5–39 min), with a target of maximum 45 min. The Danish Child and Adolescent HARDSHIP questionnaire therefore complies with the intentions of the originators, aiming at a maximal completion time of 45 min and in comparison with actual completion time.

The test-retest study showed substantial agreement between test and retest in the headache, migraine and MOH domains and questions referring to time.

The 24 h between test and retest was chosen due to practical issues at the schools involved. A test–retest period of between two days and two weeks has been recommended (Nunnally & Bernstein, 1994). Shorter periods may be associated with pupils enabling the recollection of answers, thereby inflating reliability (Nunnally & Bernstein, 1994). The accuracy of children’s ability to recall pain intensities has been shown to be high with little decrement over a 1-week period (Zonneveld et al., 1997) and a study by Van den Brink and colleagues (Van den Brink, Bandell-Hoekstra & Adu-Saad, 2001) investigating the recall bias in paediatric headache, showed that recall errors occur when children are asked to report their headaches in a retrospective questionnaire. However, unreliability in frequency estimates is of particular concern among individuals with high-frequency (chronic) headache. (Houle et al., 2013). Employing shorter recall intervals when assessing headache frequency may improve accuracy (Houle et al., 2013).

By reducing the test-retest interval the possible fluctuation of headache in children, where headaches may occur within weekly intervals and varying duration (Rho et al., 2012; Straube et al., 2013), may also influence reliability if the time interval was to be expanded by too large a time interval. We do not expect the relative short period of 24 h to be disadvantageous, as it may show a higher accuracy and recall bias may be eliminated. The Child and Adolescent HARDSHIP questionnaire (Wöber-Bingöl et al., 2014) has a total of 44 questions, which in itself may make recalling answers to specific questions difficult.

The Child and Adolescent HARDSHIP questionnaire, includes a section specifically recording a four week period. In this study, absolute agreement levels had a tendency to diminish as the recall time span lengthens. This is expected as recall bias increases over time (Stewart et al., 2000; Van den Brink, Bandell-Hoekstra & Adu-Saad, 2001; Zwart et al., 2004). The Child and Adolescent HARDSHIP questionnaire is intended to measure burden of headache in large populations and there is therefore no need to reflect the states of individuals. We therefore find the substantial reliability of the Danish version of the Child and Adolescent HARDSHIP questionnaire to be adequate, without supplementing with a diary (Zwart et al., 2004; Wöber-Bingöl et al., 2014).

The pilot study indicates that headache is a major problem among children and adolescents in Denmark. See Table 3. This pilot study clearly demonstrates the need to investigate the burden of headache among Danish schoolchildren as it seems to have a profound effect on their lives. The pilot study included a small sample size and therefore, validity of the results must be interpreted with care.

**Table 3:** Impact of headache on pupils in pilot study.

Pupils having experienced headache in pilot study (*n* = 169)	
Experienced headache in their life	161 (95.3%)
Experienced headache during the last year	129 (76.6%)
Experienced headache during the last week	83 (49.2%)
Describe headache as quite bad	79 (47%)
Have taken pills or medication during the last week	41 (24.1%)
Have left school early as a result of their headache	24 (14%)
Missed school during the last four weeks due to headache	25 (14.9%)

### Perspectives and future research

Publishing the translation of a health measurement instrument is important in order to avoid the emergence of multiple versions of the instrument and to demonstrate that the translation procedure was rigorous. A translated questionnaire also makes it possible to compare findings between and within countries (Zwart et al., 2004). The translation of the Child and Adolescent HARDSHIP Questionnaire to Danish versions was performed using rigorous methodology, adapted culturally, and face validated to a Danish context.

Previous work suggests that the Child and Adolescent HARDSHIP Questionnaire predicts substantial impact of headache in the respective age groups of 6–11 and 12–17 years of age (Wöber-Bingöl et al., 2014). The Child and Adolescent HARDSHIP questionnaire may therefore identify the prevalence of headache in Danish youth and help distinguish the types of headache from which they suffer.

## Conclusion

The translation of the Child and Adolescent HARDSHIP Questionnaire to Danish versions showed substantial test-retest study. The Child and Adolescent HARDSHIP Questionnaire may be used in a Danish context to identify the prevalence and impact of different types of headaches among children and adolescents.

Article highlights:

∙ The reliability of the Danish Child and Adolescent HARDSHIP questionnaires showed substantial agreement.
∙ The sections of the questionnaire referring to time, showed substantial agreement.
∙ The pilot study indicates that headache may be a major problem in the age groups of 6–11 and 12–17 years of age in Denmark.

## Supplemental Information

10.7717/peerj.1927/supp-1 Supplemental Information 1 Changes prompted by the pupils during the “Think aloud” interview

10.7717/peerj.1927/supp-2 Supplemental Information 2 Danish version of the HARDSHIP questionnaire age 12–17

10.7717/peerj.1927/supp-3 Supplemental Information 3 Danish version of the HARDSHIP questionnaire age 6–11

10.7717/peerj.1927/supp-4 Data S1 Raw data questionnaire 1 HARDSHIP data age 6–11.

10.7717/peerj.1927/supp-5 Data S2 Raw data questionnaire 2 HARDSHIP data age 12–17.

10.7717/peerj.1927/supp-6 Data S3 Raw data questionnaire 3 HARDSHIP test-retest.

10.7717/peerj.1927/supp-7 Supplemental Information 4 Changes prompted in the questionnaire by the interviews

## Additional Information and Declarations

### Data Availability

The following information was supplied regarding data availability: The raw data has been supplied as a Data S1, Data S2 and Data S3.
