# Supplementary material for: Translation and validation of the Child and the Adolescent HARDSHIP (Headache-attributed restriction, disability, social handicap and impaired participation) questionnaire into Danish language
Source: PeerJ. 2016 Apr 14;4:e1927. doi: 10.7717/peerj.1927 (PMC4841233; doi:10.7717/peerj.1927)
Supplement: Supplemental Information 1 [file peerj-04-1927-s001.docx]

|  | English | Problem | Pupil suggestion | Response |
| --- | --- | --- | --- | --- |
| Q8:  Which best describes your headache?  Throbbing , pulsing, pressing | “Throbbing , pulsing, pressing” | Difficulty with the description of headache | Could not find better words. No change | No change |
| Q10,11,12 | Answer possibilities:  “yes” or “no” | Some pupils wanted the possibility of “sometimes” as a possibility | No change | No change |
| Q 17  On how many days in the last week did you take medicine or pills because of headache? |  | The word “medicine” should not be there, only “pills” or “headache pills” | No change | No change |
| Q 27  I was afraid of having a headache | “afraid of” | The term “afraid of” does not relate to a headache with the pupils, but more “as afraid of” something that can harm | Change the term “afraid” with “often think about” | No change |
| **Q 31**  **I was able to cope well with my headaches** | **“cope”** | **Difficulty with the Danish translation “haandtere”** | **Change “haandtere” with “styre”** | **Changing the word ”haandtere” with “afhjaelpe”** |
| **Q 33-44**  **Please think about your life in the four weeks to answer these questions.** |  | **Difficulty in seeing these questions as questions not related to headache but life in general.** |  | **Response:**  **More instruction prior to Q33 - 44, clarifying this situation**  **”Tænk på dit liv i de sidste 4 uger når du besvarer de næste spoergsmål. Det handler Ikke om din hovedpine, men om dit liv generelt”** |
| Q 34  I was tired and worn-out | ”worn-out” and ”tired” | Difficulty in understanding ”worn-out” | Only use ”tired” | Response:  No change |
| **Q 40**  **I felt pleased with myself** | **”pleased with myself”** | **Difficulty in understanding the Danish translation: “ Jeg har vaeret tilfreds med mig selv”** | **Change the Danish wording to:**  **“Jeg har været glad for mig selv”** | **Response:**  **Change of wording to:**  **“Jeg har været glad for mig selv”** |
| **Q 42**  **I got along with my friends** | **”got along with my friends”** | **Difficulty with the Danish translation and the word “fint”** | **Change “fint” with “godt”** | **Response:**  **Change of wording from “fint” to “godt”** |

Supplementary material:

Changes prompted by the pupils during the “Think aloud” interview.
