# Supplementary material for: Translation and validation of the Child and the Adolescent HARDSHIP (Headache-attributed restriction, disability, social handicap and impaired participation) questionnaire into Danish language
Source: PeerJ. 2016 Apr 14;4:e1927. doi: 10.7717/peerj.1927 (PMC4841233; doi:10.7717/peerj.1927)
Supplement: Supplemental Information 2 [file peerj-04-1927-s002.pdf]

Lifting The Burden
The Global Campaign against Headache

Global kampagne mod hovedpine

Et selv-administrerede spørgeskema til børn i aldersgruppen 12 -17år

Formulartitel

1.Hvilken dato er det i dag?*
Dag Måned 2015

2. Hvor gammel er du?*.Marker hvor gammel du er

- 12 år
- 13 år
- 14 år
- 15 år
- 16 år
- 17 år

3. Er du en en dreng eller en pige ?*

- Dreng
- Pige

Screeningsspørsgsmål

4. Har du nogensinde haft hovedpine?*Skal udfyldes

- Ja
- nej

5. Har du haft hovedpine indenfor det sidste år ?
*.Vælg ja eller nej - Har du svaret nej - fortsættes direkte til spørgsmål 33Janej

Diagnosticerende spørgsmål

Nedenstående spørgsmål skal beskrive din hovedpine

6. Hvor lang tid plejer din hovedpine at vare?*Sæt kryds

- under 1 time
- 1-2 timer
- 2-4 timer
- mere end 4 timer

7. Hvor slem er din hovedpine?*

- ikke slemt
- slem
- meget slem

8. Hvordan beskrives bedst din hovedpine?* Sæt kryds ved den, der beskriver det bedst

- Dunkende eller pulserende - som et hjerteslag
- Trykkende

9. Mærker du normalt din hovedpine i den ene side af hovedet , i midten eller i begge sider?*
Sæt kryds

- Kun én side
- I midten
- Begge sider

10. Får bevægelse (f.eks gang eller at gå på trapper) din hovedpine til at blive værre?*
Sæt kryds

- nej
- ja

11. Undgår du bevægelse, (f.eks gang eller at gå på trapper), når du har hovedpine*
Sæt kryds

- nej
- ja

12. Føler du dig syg, når du har hovedpine? (f.eks som om du skal kaste op)*
Sæt kryds

- nej
- ja

13. Er du syg, når du har hovedpine (kaster du f.eks op)* Sæt er kryds

- nej
- ja

14. Kan du bedst lide at være i mørke, når du har hovedpine?* sæt kryds

- nej
- ja

15. Kan du bedst lide, at der er stille omkring dig , når du har hovedpine* sæt kryds

- nej
- ja

Spørgsmål om påvirkning/konsekvenser

De næste spørgsmål handler om, hvordan hovedpinen påvirker din hverdag.

De første to spørgsmål handler om den sidste uge

16. Hvor mange dage har du haft hovedpine i løbet af den sidste uge*S
Skriv antal dage mellem 0 og 7

17. Hvor mange dage i løbet af den sidste uge har du taget medicin eller piller på grund af din hovedpine?*S
Skriv antal dage mellem 0 og 7

De næste spørgsmål omhandler de sidste fire uger.

18. Hvor mange dage i løbet af de sidste fire uger har du haft hovedpine ?*S
Skriv et tal mellem 0 og 28

19. Hvor mange dage i løbet af de sidste fire uger har du taget medicin eller piller på grund af din hovedpine*SkSkriv venligst et tal mellem 0 og 28

20. Hvor mange dage, i løbet af de sidste fire uger, har du ikke været i skole på grund af hovedpine?*SIndtast venligst et tal mellem 0 og 20

21. Hvor mange dage i løbet af de sidste fire uger er du taget tidligere hjem fra skole på grund af hovedpine?*SSkriv venligst et tal mellem 0 og 20

22. Hvor mange dage i løbet af de sidste fire uger, har du ikke kunne gør ting du gerne ville på grund af din hovedpine? *Skriv venligst et tal mellem 0 og 28

23. Har dine forældre i løbet af de sidste fire uger, været hjemme fordi du havde hovedpine?*Skal
Hvis ja, skriv hvor mange dage ialt

- ja
- nej
- Andet:

Spørgsmål om i går

24. Havde du hovedpine i går ?* Skal udfyldes Hvis du svarer nej, fortsæt da derefter til spørgsmål 27

- Nej
- Ja

25. Hvor slemt var det ?* Marker

- Ikke slemt
- Ret slemt
- Meget slemt

26. Gik du glip af skolen i går ?* Marker dit svar

- Nej
- ja, jeg tog tidligere hjem
- ja, jeg blev hjemme

Flere spørgsmål om påvirkning/konsekvens

Tænk på dit liv de sidste fire uger, når du besvarer de næste spørgsmål.

27. Jeg har været bekymret for at få hovedpine* Marker dit svar

- Aldrig
- nogle gange
- Ofte
- altid

28. Jeg måtte ikke lave noget for mine forældre, fordi jeg havde hovedpine* Marker dit svar

- aldrig
- nogle gange
- ofte
- altid

29. Jeg kunne ikke koncentrere mig, fordi jeg havde hovedpine* Marker dit svar

- aldrig
- nogle gange
- ofte
- altid

30. Jeg har været ked af det på grund af min hovedpine* Marker dit svar

- aldrig
- nogle gange
- ofte
- altid

31. Jeg har været i stand til at afhjælpe min hovedpine* Marker dit svar

- aldrig
- nogle gange
- ofte
- altid

32. Jeg ville ikke have, at nogen skulle opdage, at jeg havde hovedpine Marker dit svar

- aldrig
- nogen gange
- ofte
- altid

De resterende spørgsmål SKAL alle svare på.

Spørgsmål om livskvalitet (med og uden hovedpine)

Tænk på dit liv i de sidste 4 uger når du besvarer de næste spørgsmål. Det handler Ikke om hovedpine, men om dit liv generelt

33. Jeg har følt mig syg* Skal udfyldes Marker dit svar

- aldrig
- nogle gange
- ofte
- altid

34. Jeg har været træt og udmattet* Skal udfyldes Marker dit svar

- aldrig
- nogle gange
- ofte
- altid

35. Jeg har haft en masse energi* Skal udfyldes Marker dit svar

- aldrig
- nogle gange
- ofte
- altid

36. Jeg har haft det sjovt og har grinet meget*Skal udfyldes Marker dit svar

- aldrig
- nogle gange
- ofte
- altid

37. Jeg har kedet mig*Skal udfyldes Marker dit svar

- aldrig
- nogle gange
- ofte
- altid

38. Jeg har følt mig alene*Skal udfyldes Marker dit svar

- aldrig
- nogle gange
- ofte
- altid

39. Jeg har været bange*Skal udfyldes Marker dit svar

- aldrig
- nogle gange
- ofte
- altid

40. Jeg har været glad for mig selv*Skal udfyldes Marker dit svar

- aldrig
- nogle gange
- ofte
- altid

41. Jeg har haft det godt derhjemme*Skal udfyldes Marker dit svar

- aldrig
- nogle gange
- ofte
- altid

42. Jeg har haft det fint med mine venner*Skal udfyldes Marker dit svar

- aldrig
- nogle gange
- ofte
- altid

43. Jeg har følt mig anderledes i forhold til andre børn Marker dit svar

- aldrig
- nogle gange
- ofte
- altid

44. Det har været nemt at lave mine lektier* Skal udfyldes Marker dit svar

- aldrig
- nogle gange
- ofte
- altid

Så er spørgeskemaet slut. Husk at trykke på SEND.

Mange tak fordi du ville være med til at besvare det.
God dag
