# Supplementary material for: Translation and validation of the Child and the Adolescent HARDSHIP (Headache-attributed restriction, disability, social handicap and impaired participation) questionnaire into Danish language
Source: PeerJ. 2016 Apr 14;4:e1927. doi: 10.7717/peerj.1927 (PMC4841233; doi:10.7717/peerj.1927)
Supplement: Supplemental Information 4 [file peerj-04-1927-s007.docx]

| Changes prompted in the questionnaire by the interviews | | | | |
| --- | --- | --- | --- | --- |
|  | English | Problem | Pupil suggestion | Response |
| Q8:  Which best describes your headache?  Throbbing , pulsing, pressing | “Throbbing , pulsing, pressing” | Difficulty with the description of headache | Could not find better words. No change | No change |
| Q10,11,12 | Answer possibilities:  “yes” or “no” | Some pupils had a difficulty in answering “yes” or “no” | Some pupils would like the choice of “sometimes” | No change |
| Q 17  On how many days in the last week did you take medicine or pills because of headache? |  | The word “medicine” confused | The word “medicine” should not be included, only “pills” or “headache pills” | No change |
| Q 27  I was afraid of having a headache | “afraid of” | The term “afraid of” does not relate to a headache with the pupils, but more “as afraid of” something that can harm | Change the term “afraid” with “often think about” | No change |
| Q 31  I was able to cope well with my headaches | “cope” | Difficulty with the Danish translation “haandtere” | Change “haandtere” with “kontrollere” | Changing the word ”haandtere” with “kontrollere” |
| Q 33-44  Please think about your life in the four weeks to answer these questions. |  | Difficulty in seeing these questions as questions not related to headache but life in general. | Specify | Response:  Clarifying instruction prior to Q33 - 44,  ” Svar på, hvordan har du generelt haft det de sidste fire uger. (både med eller uden hovedpine)” |
| Q 34  I was tired and worn-out | ”worn-out” and ”tired” | Difficulty in understanding ”worn-out” | Only use ”tired” | Response:  Change to a synomym:  “udkoert” |
| Q 40  I felt pleased with myself | ”pleased with myself” | Difficulty in understanding the Danish translation: “ Jeg har vaeret tilfreds med mig selv” | Change the Danish wording to:  “Jeg har været glad for mig selv” | Response:  Change of wording to:  “Jeg har været glad for mig selv” |
| Q 42  I got along with my friends | ”got along with my friends” | Difficulty with the Danisk translation and the word “fint” | Change “fint” with “godt” | Response:  Change of wording from “fint” to “godt” |

Table 2 showing the actions taken after the Think Aloud usability study.
